# Phytoestrogen consumption from foods and supplements and epithelial ovarian cancer risk: a population-based case control study

**DOI:** 10.1186/1472-6874-11-40

**Published:** 2011-09-23

**Authors:** Elisa V Bandera, Melony King, Urmila Chandran, Lisa E Paddock, Lorna Rodriguez-Rodriguez, Sara H Olson

**Affiliations:** 1 Cancer Prevention and Control Program/Division of Surgical Oncology, The Cancer Institute of New Jersey, Robert Wood Johnson Medical School, New Brunswick, NJ, USA; 2 School of Public Health, University of Medicine and Dentistry of New Jersey, Piscataway, NJ, USA; 3 New Jersey Department of Health and Senior Services, Trenton, NJ, USA; 4 Department of Obstetrics and Gynecology and Reproductive Sciences, Division of Gynecologic Oncology, The Cancer Institute of New Jersey, UMDNJ/Robert Wood Johnson Medical School, New Brunswick, NJ, USA; 5 Memorial Sloan-Kettering Cancer Center, New York, NY, USA

## Abstract

**Background:**

While there is extensive literature evaluating the impact of phytoestrogen consumption on breast cancer risk, its role on ovarian cancer has received little attention.

**Methods:**

We conducted a population-based case-control study to evaluate phytoestrogen intake from foods and supplements and epithelial ovarian cancer risk. Cases were identified in six counties in New Jersey through the New Jersey State Cancer Registry. Controls were identified by random digit dialing, CMS (Centers for Medicare and Medicaid Service) lists, and area sampling. A total of 205 cases and 390 controls were included in analyses. Unconditional logistic regression analyses were conducted to examine associations with total phytoestrogens, as well as isoflavones (daidzein, genistein, formononetin, and glycitein), lignans (matairesinol, lariciresinol, pinoresinol, secoisolariciresinol), and coumestrol.

**Results:**

No statistically significant associations were found with any of the phytoestrogens under evaluation. However, there was a suggestion of an inverse association with total phytoestrogen consumption (from foods and supplements), with an odds ratio (OR) of 0.62 (95% CI: 0.38-1.00; p for trend: 0.04) for the highest vs. lowest tertile of consumption, after adjusting for reproductive covariates, age, race, education, BMI, and total energy. Further adjustment for smoking and physical activity attenuated risk estimates (OR: 0.66; 95% CI: 0.41-1.08). There was little evidence of an inverse association for isoflavones, lignans, or coumestrol.

**Conclusions:**

This study provided some suggestion that phytoestrogen consumption may decrease ovarian cancer risk, although results did not reach statistical significance.

## Background

Cancer of the ovary is the second most common gynecologic cancer and the leading cause of death from gynecologic malignancies [1]. While the etiology of ovarian cancer is not well understood, a protective effect of oral contraceptive use and higher parity is widely accepted [2]. The main theories proposed to explain ovarian pathogenesis are "incessant ovulation" proposed by Fathalla [3] and excessive gonadotropin stimulation of the ovarian epithelium proposed by Stadel [4]. Proponents of the former theory argue that ovulation results in minor trauma to the ovarian epithelium leading to rapid proliferation to repair the ovulatory wound. Abnormal proliferation or malignant transformation may result from excess stimulation by hormonal factors, such as estrogen-rich follicular fluid after ovulation or excessive gonadotropin levels leading to stimulation by estrogens or estrogen precursors [5]. There has also been a growing interest in the role of inflammation on ovarian cancer. Proponents of this theory argue that repeated ovulation causes inflammation, which leads to stress in the ovarian epithelial surface cells, predisposing them to genetic damage and malignant transformation [6]. Based on these theories and what we know about ovarian cancer etiology, factors capable of affecting gonadotropins or estrogens, including their synthesis, metabolism, actions, or regulation, can potentially affect ovarian cancer risk. The experimental evidence suggests that phytoestrogens may affect gonadotropin and estrogen levels [7], as well as cytokine production [8].

Phytoestrogens are non-steroidal plant-derived compounds, with a similar structure as endogenous estrogens, and capable of showing both estrogenic and antiestrogenic effects [9,10]. Main dietary phytoestrogens are isoflavones (found mainly in soy products) and lignans, more widely distributed in the Western diet (found in flaxseed, grain/breads, nuts, coffee, tea, fruits, and vegetables) [11]. Plant lignans are transformed by the intestinal microflora into the enterolignans, enterodiol and enterolactone, which are believed to be more physiologically active than their precursors [12]. For years, only two plant lignans were considered enterolignan precursors, secoisolariciresinol and matairesinol. However, other plant lignans, lariciresinol and pinoresinol, have now been shown to have high conversion rates into enterolignans, while food content on these lignans have only recently become available [13].

While there is an extensive literature evaluating the impact of phytoestrogen consumption on breast cancer risk, its role on ovarian cancer has received little attention [7]. Out of six studies that have examined the role of main phytoestrogens or foods high in phytoestrogens and ovarian cancer, five studies tended to suggest an inverse association [14-20]. In contrast, a recent cohort study in Sweden failed to find an association with phytoestrogen intake [21]. A meta-analysis including the four studies that evaluated soy [15-18] also reported reduced risk [22].

We conducted a population-based case-control study, the *NJ Ovarian Cancer Study*, specifically designed to evaluate phytoestrogens and ovarian cancer risk in New Jersey, a population characterized by large ethnic diversity. Consumption of foods high in phytoestrogens not included in the Block food frequency questionnaire (FFQ) (see Appendix 1) was ascertained and a detailed phytoestrogen composition database [23] was used to derive intake levels of all the major isoflavones and lignans, as well as total phytoestrogens. Furthermore, we ascertained and examined the role of phytoestrogen/isoflavone supplements on ovarian cancer risk.

## Methods

The *NJ Ovarian Cancer Study *has been described in detail elsewhere [24]. In summary, the study builds upon the EDGE Study (Estrogen, Diet, Genetics, and Endometrial Cancer), a population-based case-control study based in New Jersey [25,26]. We used the controls from the EDGE Study and added ovarian cancer cases to form a new case-control study. Same eligibility criteria and methods were carefully implemented in the case and the control groups. Cases were newly diagnosed histologically confirmed cases of invasive epithelial ovarian cancer identified between January 2004 and May 2008 through rapid case ascertainment by the New Jersey State Cancer Registry (NJSCR), a population-based SEER cancer registry that has collected data since 1978. Women older than 21 years, able to understand English or Spanish, and residing in one of six New Jersey counties (Bergen, Essex, Hudson, Middlesex, Morris, and Union) were eligible to participate. A total of 682 eligible cases were initially identified. Of them, 70 cases were not contacted because they were deceased (n = 61) or their physicians advised us not to contact them (n = 9). Additionally 119 people were ineligible because they could not be reached, no longer met eligibility requirements, there was a communication barrier, or they reported some other medical condition that precluded participation. Of the 493 remaining cases, 252 consented to participate (51%) and 233 completed the interview (47%).

The control group was the same as in the EDGE Study, described in detail elsewhere [24-27]. In brief, controls had the same eligibility criteria as the cases except that women with a history of hysterectomy and/or bilateral oophorectomy were excluded from the analysis. Random digit dialing was employed to recruit women under 65 years of whom 355 were eligible to participate and 175 completed the interview. Women older than 65 years were located through random selection by using lists purchased from the Center for Medicare and Medicaid Services (CMS); 68 women from this source completed the study. Finally, an area sampling approach identified 524 women older than 55 years who were eligible to participate, out of whom 224 completed the study. Overall, a total of 467 (approximately 40%) controls from the three sources completed the study.

Informed consent was obtained from all participants, and the study was approved by the Institutional Review Boards at Robert Wood Johnson Medical School, Memorial Sloan-Kettering Cancer Center, and the New Jersey Department of Health and Senior Services.

### Data Collection

We used the same procedures in the *NJ Ovarian Cancer Study *and the EDGE Study to standardize data collection in cases and controls. Interviewers were trained using the same procedures and same training manual. Interviews, conducted by telephone for most respondents, covered established and suspected risk factors for ovarian cancer. In addition to the interview, participants were mailed a package with instructions for providing buccal specimens and waist and hip circumference measurements and the Block 98.2 food frequency questionnaire (FFQ). Participants were instructed to report their usual intake of the food items in the questionnaire during the six months before diagnosis (for cases) or the date of the interview (for controls). Two hundred and five (88%) cases and 398 controls (85%) returned the FFQ. The participants who returned the FFQ tended to be older, but there were no significant differences in education, oral contraceptive use, hormone replacement therapy use, tubal ligation or family history of ovarian cancer (data not shown). Eight of the controls were excluded because both of their ovaries had been removed, placing them at negligible risk of developing ovarian cancer, resulting in 390 controls being included in analyses.

The Block 98.2 FFQ (NutritionQuest, Berkeley, CA) includes 110 food items and was developed using the NHANES (National Health and Nutrition Examination Survey) III dietary recall data. It also includes questions on portion size for each food, and pictures are provided to facilitate estimation. The questionnaire includes a variety of foods containing phytoestrogens, such as several kinds of beans, tofu, soymilk, canned tuna fish, meat substitutes (e.g., veggie burgers, veggie chicken), and whole wheat bread. To supplement the list of foods, we added one page with 21 additional food items, based on the LACE questionnaire [28] and including other food items that have been identified as important sources of phytoestrogens [29]. The additional foods in the supplemental page that we added, and that are not included in the Block 98.2 questionnaire, are listed in Appendix 1. We also asked about the use of phytoestrogen/soy supplements including frequency and duration of use. NutritionQuest provided nutrient calculations using the USDA Nutrient Database for Standard Reference. For phytoestrogen calculations we used a Canadian database with detailed analyses of phytoestrogen content of foods, including detailed values for lignans [23]. Given the global food trading, we do not expect major differences in lignan composition between foods available in the United States and Canada.

### Statistical Analysis

We evaluated the association of ovarian cancer risk with total phytoestrogens, as well as with major phytoestrogen groups (isoflavones, lignans, and coumestan) and specific phytoestrogens. This included isoflavones (daidzein, genistein, formononetin, and glycitein, and total isoflavones calculated by summing those four isoflavones), lignans (matairesinol, lariciresinol, pinoresinol, secoisolariciresinol and total lignans calculated by summing the four lignans), and coumestrol. Total phytoestrogen consumption was computed by adding total isoflavones, total lignans and coumestrol. Age-adjusted means were compared using ANCOVA. Participants were categorized into tertiles of intake based on the distribution of intake in controls. Odds ratios (OR) and 95% confidence intervals (CI) were estimated by unconditional multiple logistic regression. Tests for trend were derived by assigning the median value to each tertile. Potential confounding variables considered were age; education (high school or less, college, graduate school); race; age at menarche (continuous); menopausal status; parity (0-1, 2, ≥ 3); oral contraceptive use (ever, never used); use of hormone replacement therapy (HRT) (never used any HRT, used unopposed estrogen only, used combined therapy, i.e., estrogen and progesterone); tubal ligation, BMI, calculated as weight (in kg) divided by height (in m^2^); total energy intake (as a continuous variable), smoking status; alcohol use (g/1000 kcal); and physical activity in metabolic equivalents (METs) for reported average hours per week of strenuous or moderate recreational activities. We adjusted for total energy intake using the multivariate nutrient density model, by computing nutrient density for each variable expressed in mcg (or mg) per 1000 kcal of intake and including total calories as a continuous variable in the model [30]. We repeated analyses adjusting for alcohol consumption, but estimates essentially did not change. SAS version 9.2 (SAS Institute, Cary NC) was used for analysis.

## Results

Main characteristics of participants in the study are shown in Table 1. The distribution according to main factors affecting the risk of the disease was similar to that reported in other studies, with higher parity and having had a tubal ligation showing a particularly strong inverse association.

**Table 1:** Selected characteristics of women participating in the NJ Ovarian Cancer Study.

	Cases (*n *= 205) n (%)		Controls (*n *= 391) n (%)		OR (95% CI)*
**Education**					
High school or less	61	(29.8)	133	(34.0)	1.00 (Ref)
College	93	(45.4)	159	(40.7)	0.90 (0.59-1.38)
Graduate school	51	(24.9)	99	(25.3)	0.76 (0.47-1.24)
**Race/ethnicity**					
White	179	(87.3)	344	(88.4)	1.00 (Ref)
Black	9	(4.4)	17	(4.4)	1.02 (0.42-2.44)
Other	8	(3.9)	17	(4.4)	0.82 (0.33-1.99)
Hispanic (any race)	9	(4.4)	11	(2.8)	1.13 (0.44-2.92)
**BMI**					
Underweight (< 18.5)	1	(0.5)	1	(0.3)	1.02 (0.06-17.31)
Normal (18.5-25)	90	(43.9)	180	(46.4)	1.00 (Ref)
Overweight (25-29.9)	54	(26.3)	122	(31.4)	1.07 (0.69-1.65)
Obese (30-34.9)	36	(17.6)	59	(15.2)	1.39 (0.83-2.32)
Very obese (≥ 35)	24	(11.7)	26	(6.7)	1.54 (0.81-2.89)
**Parity**					
0 - 1	97	(47.3)	92	(23.5)	1.00 (Ref)
2	60	(29.3)	137	(35.0)	0.45 (0.29-0.69)
≥ 3	48	(23.4)	162	(41.4)	0.42 (0.26-0.66)
**Smoking status**					
Never	108	(52.7)	204	(52.2)	1.00 (Ref)
Past	78	(38.1)	149	(38.1)	1.12 (0.76-1.64)
Current	19	(9.3)	38	(9.7)	0.87 (0.46-1.62)
**Oral contraceptive use**					
Never	85	(41.5)	193	(49.4)	1.00 (Ref)
Ever	120	(58.5)	198	(50.6)	0.88 (0.61-1.28)
**Use of HRT**					
Never	159	(77.6)	285	(72.9)	1.00 (Ref)
Unopposed E only	22	(10.7)	34	(8.7)	1.56 (0.86-2.84)
Any combined HRT	24	(11.7)	72	(18.4)	0.63 (0.38-1.06)
**Age at menarche**					
> 13	41	(20.1)	99	(25.4)	0.81 (0.51-1.27)
12-13	117	(57.4)	200	(51.3)	1.00 (Ref)
≤11	46	(22.6)	91	(23.3)	0.75 (0.48-1.17)
**Menopause status**					
Premenopausal	71	(34.6)	49	(12.5)	1.51 (0.85-2.69)
Postmenopausal					
Age at menopause					
< 40	5	(2.4)	14	(3.6)	0.77 (0.26-2.31)
41-54	86	(42.0)	239	(61.3)	1.00 (Ref)
≥ 55	12	(5.9)	37	(9.5)	0.99 (0.48-2.01)
Unknown	31	(15.1)	52	(13.3)	1.52 (0.91-2.56)
**Tubal Ligation**					
No	175	(85.4)	315	(80.6)	1.00 (Ref)
Yes	30	(14.6)	76	(19.4)	0.59 (0.36-0.94)
**First relative with ovarian cancer**					
No	195	(95.1)	377	(96.4)	1.00 (Ref)
Yes	10	(4.9)	14	(3.6)	1.32 (0.55-3.17)

*Adjusted for age

Age-adjusted means for phytoestrogen and total caloric intake in cases and controls are compared in Table 2. Total isoflavones, total lignans, and total phytoestrogen consumption, both from food only and from food and supplements, were lower in cases than controls, but none of the differences were statistically significant. As expected, the major source of phytoestrogen consumption in this population was lignans. Use of isoflavone/phytoestrogen supplements was low in this population, with only 5.4% of the cases and 4.9% of the controls reporting to ever use them (p value: 0.84). Similarly, use of soy powders was reported by only 6.3% of the cases and 3.9% of the controls (p value: 0.19) (data not shown).

**Table 2:** Age-adjusted mean phytoestrogen and caloric intake in cases and controls.

	From food		
Compound (mcg/1000 kcal)	Cases Mean (SE)	Controls Mean (SE)	p value
Total isoflavones	805.67 (182.55)	1154.43 (130.17)	0.13
Daidzein	332.61 (72.92)	451.45 (51.99)	0.20
Genistein	438.91 (105.10)	657.37 (74.94)	0.10
Formononetin	6.95 (0.49)	6.70 (0.35)	0.69
Glycitein	27.10 (6.92)	38.78 (4.93)	0.18
			
Total lignans	569.26 (40.48)	623.90 (28.86)	0.28
Matairesinol	3.90 (0.13)	3.77 (0.10)	0.44
Lariciresinol	34.21 (1.33)	37.14 (0.95)	0.08
Pinoresinol	24.59 (1.13)	26.12 (0.81)	0.28
Secoisolariciresinol	506.56 (40.22)	556.88 (28.68)	0.32
			
Coumestrol	0.85 (0.04)	0.87 (0.03)	0.68
			
Total phytoestrogens	1375.55 (188.90)	1778.97 (134.69)	0.09
			
Total calories (kcal)	1598.9 (47.97)	1602.3 (34.15)	0.95

	**From food and supplements**		
Total isoflavones	1513.06 (437.96)	2230.59 (312.28)	0.19
Total phytoestrogens	2082.94 (441.70)	2855.13 (314.95)	0.16

Risk estimates for total phytoestrogens, isoflavones, and lignans are shown in Table 3. After adjusting for age, education, race, major reproductive risk factors, BMI and total calories, there was a suggestion of a decreased risk with phytoestrogen consumption, with an OR of 0.62 (95% CI: 0.38-1.00; p for trend: 0.04) for the highest tertile of total phytoestrogen intake from foods and supplements compared to the lowest. Further adjustment for smoking and physical activity attenuated estimates, with an OR of 0.66 (95% CI: 0.41- 1.08; p for trend: 0.11). Adding alcohol intake to the model did not change risk estimates (data not shown). There was little evidence of an association for isoflavones or lignans, as shown in Table 3. The OR for the highest vs. the lowest tertile of total lignan intake was 1.10 (95% CI: 0.68-1.79). There was no evidence of an association for coumestrol (data not shown).

**Table 3:** Isoflavone and total phytoestrogen intake and ovarian cancer risk in the NJ Ovarian Cancer Study.

	Cases (n)	Controls (n)	OR1	95% CI	OR2	95% CI
**FROM FOOD**						
**Daidzein **(mcg/1000 kcal)						
1 (< 20.25)	58	129	1.00		1.00	
2 (20.25-144.07)	86	131	1.29	0.81-2.06	1.34	0.83-2.16
3 (≥ 144.08)	61	130	0.80	0.48-1.31	0.88	0.53-1.46
*p *for trend			0.10		0.21	
						
**Genistein **(mcg/1000 kcal)						
1 (< 40.46)	62	129	1.00		1.00	
2 (40.46-247.85)	82	132	1.17	0.74-1.86	1.22	0.76-1.96
3 (≥ 247.86)	61	129	0.75	0.46-1.23	0.83	0.50-1.38
*p *for trend			0.09		0.21	
						
**Formononetin **(mcg/1000 kcal)						
1 (< 3.90)	78	130	1.00		1.00	
2 (3.90-6.80)	75	130	1.01	0.64-1.59	1.02	0.64-1.62
3 (≥ 6.81)	52	130	0.69	0.42-1.14	0.72	0.43-1.22
*p *for trend			0.11		0.17	
						
**Glycitein **(mcg/1000 kcal)						
1 (< 2.14)	67	130	1.00		1.00	
2 (2.14-9.17)	72	131	0.98	0.62-1.57	0.95	0.59-1.53
3 (≥ 9.18)	66	129	0.74	0.46-1.21	0.80	0.48-1.33
*p *for trend			0.18		0.38	
**Total Isoflavones **(mcg/1000 kcal)						
1 (< 70.06)	61	129	1.00		1.00	
2 (70.06-404.66)	83	132	1.20	0.76-1.92	1.24	0.77-2.00
3 (≥ 404.67)	61	129	0.78	0.48-1.27	0.86	0.52-1.42
*p *for trend			0.11		0.24	
**Matairesinol **(mcg/1000 kcal)						
1 (< 2.82)	69	129	1.00		1.00	
2 (2.82-4.22)	69	131	1.05	0.66-1.67	1.00	0.68-1.78
3 (≥ 4.23)	67	130	1.41	0.88-2.27	1.58	0.97-2.58
*p *for trend			0.14		0.06	
						
**Lariciresinol **(mcg/1000 kcal)						
1 (< 27.17)	80	130	1.00		1.00	
2 (27.17-40.01)	68	131	0.99	0.62-1.57	1.04	0.65-1.66
3 (≥ 40.02)	57	129	0.98	0.60-1.59	1.06	0.64-1.74
*p *for trend			0.93		0.83	
						
**Pinoresinol **(mcg/1000 kcal)						
1 (< 18.08)	84	129	1.00		1.00	
2 (18.08-28.50)	62	132	0.76	0.48-1.20	0.80	0.50-1.29
3 (≥ 28.51)	59	129	0.92	0.58-1.48	0.97	0.59-1.58
*p *for trend			0.78		0.94	
						
**Secoisolariciresinol **mcg/1000 kcal)						
1 (< 193.64)	63	130	1.00		1.00	
2 (193.64-624.92)	84	131	1.27	0.80-2.01	1.33	0.83-2.13
3 (≥ 624.93)	58	129	1.12	0.69-1.80	1.11	0.68-1.81
*p *for trend			0.81		0.90	
						
**Total lignans **(mcg/1000 kcal)						
1 (< 271.22)	67	129	1.00		1.00	
2 (271.22-704.75)	79	131	1.21	0.77-1.91	1.23	0.77-1.97
3 (≥ 704.76)	59	130	1.12	0.70-1.79	1.10	0.68-1.79
*p *for trend			0.77		0.84	
**Total phytoestrogens **(mcg/1000 kcal)						
1 (< 532.28)	80	130	1.00		1.00	
2 (532.28-1287.81)	68	131	0.79	0.50-1.25	0.73	0.46-1.16
3 (≥ 1287.82)	57	129	0.68	0.42-1.08	0.73	0.45-1.19
*p *for trend			0.12		0.29	
						

**FROM FOOD and SUPPLEMENTS**						
**Daidzein **(mcg/1000 kcal)						
1 (< 21.40)	59	130	1.00		1.00	
2 (21.40-167.32)	90	131	1.35	0.85-2.15	1.40	0.87-2.26
3 (≥ 167.33)	56	129	0.73	0.44-1.21	0.82	0.49-1.38
*p *for trend			0.03		0.10	
						
**Genistein **(mcg/1000 kcal)						
1 (< 41.14)	60	130	1.00		1.00	
2 (41.14-275.32)	86	131	1.25	0.78-1.99	1.31	0.82-2.11
3 (≥ 275.33)	59	129	0.82	0.50-1.34	0.92	0.55-1.54
*p *for trend			0.14		0.34	
						
**Glycitein **(mcg/1000 kcal)						
1 (< 2.17)	67	129	1.00		1.00	
2 (2.17-11.64)	77	132	1.00	0.63-1.58	0.96	0.60-1.54
3 (≥ 11.65)	61	129	0.70	0.43-1.15	0.76	0.46-1.25
*p *for trend			0.11		0.24	
**Total Isoflavones **(mcg/1000 kcal)						
1 (< 71.80)	60	130	1.00		1.00	
2 (71.80-497.97)	86	130	1.29	0.81-2.05	1.35	0.84-2.17
3 (≥ 497.98)	59	130	0.78	0.47-1.28	0.88	0.53-1.46
*p *for trend			0.08		0.21	
**Total phytoestrogens **(mcg/1000 kcal)						
1 (< 534.35)	79	130	1.00		1.00	
2 (534.35-1395.56)	74	130	0.88	0.56-1.38	0.82	0.52-1.30
3 (≥ 1395.57)	52	130	0.62	0.38-1.00	0.66	0.41-1.08
*p *for trend			0.04		0.11	

OR1: adjusted for age (continuous), education, race, age at menarche (continuous), menopausal status, parity, OC use, HRT use, BMI (continuous), tubal ligation, and total calories (see methods for more details).

OR2: further adjusted for physical activity (METs for average hours of strenuous or moderate recreational activities per week) and smoking (smoking status and pack-years for current & past smokers). Further adjustment for alcohol intake did not change risk estimates

We further explored the association with the major food sources of isoflavones, soy products (data not shown). Intake was low in this population and, therefore, we were only able to categorize women into ever vs. never consumers for individual soy foods. There was a suggestion of a decreased risk for tofu and total soy products. However, after including smoking and physical activity in the model with all the other major risk factors, the confidence interval included one. For total soy products, the OR for those consuming more than one cup per month of soy products compared to never users was 0.71 (95% CI: 0.42-1.20).

## Discussion

In this study we found a suggestion of an inverse association between phytoestrogen consumption and ovarian cancer risk. However, confidence intervals included the null value after adjusting for covariates, including physical activity and smoking. Estimates were stronger when combined intake from foods and supplements was evaluated. Similarly, there was some suggestion of an inverse association with tofu and combined soy product consumption, but confidence intervals included one.

Few epidemiologic studies have evaluated the association between phytoestrogens and ovarian cancer and, to our knowledge, this is the first population-based study to undertake a detailed analysis of lignans, isoflavones, and total phytoestrogens in relation to ovarian cancer risk in the United States, as well as attempting to compute total phytoestrogens from foods and supplements. Total isoflavone intake was previously evaluated in two cohort studies in California [16] and Sweden [21] and two hospital-based studies conducted in China [15] and Italy [18]. Three of these studies [15,16,18] found an inverse association with isoflavone intake, with approximately 50% reduced risk for the highest category of consumption compared to the lowest. However, in the cohort study in Sweden [21], like in our study, there was little evidence of an association. Tofu was also evaluated in the California Teachers Cohort [16] and in the Japan Collaborative Cohort (JACC) Study [17]. Similar to our findings, both studies found risk estimates below one, but the confidence interval included the null. Tofu and meat substitutes were uncommonly used in both our study and in the California Teachers Cohort, and that may have affected our ability to detect an association. In the Chinese hospital-based case-control study [15], which had a wider range of intake, soy foods were also found to decrease ovarian cancer risk, with an OR of 0.50 (95% CI: 0.31-0.82) [15].

We are aware of two other studies reporting on lignan intake and ovarian cancer risk, with conflicting results. The first is a case-control study in Western New York, which evaluated two lignans combined, secoisolariciresinol and matairesinol, and found a strong inverse association with intake [20]. The second study, is a cohort study in Sweden [21] which, like ours, found no indication of an association with lignan consumption. Lignan calculations were computed using different databases in the three studies and this, together with possibly different food sources, may have explained, at least in part, the discrepant findings.

Several mechanisms have been postulated to explain the potential protective effect of soy and phytoestrogens on ovarian carcinogenesis. They have been shown to inhibit enzymes that synthesize and metabolize estrogens and increase sex hormone-binding globulin synthesis [22]. In addition, they are capable of binding to the estrogen receptor (ER), particularly ER beta, which has been involved in the differentiation of proliferating tissue [22]. Other anticarcinogenic properties that have been attributed to phytoestrogens include inhibiting tumor angiogenesis, cell proliferation, tyrosine kinase, and topoisomerase II [22].

Our study is subject to the limitations of case-control studies, such as potential recall bias, as cases may report or recall dietary intake in a different way than controls. However, because the relationship of ovarian cancer with soy foods is largely unknown, and lignans are widely distributed in the diet, this is unlikely. The response rate in our study was low. However, it is well known that participation rates in population-based studies have been decreasing over the past years, and rates around 50% are not unusual, particularly among controls [31]. To evaluate possible selection bias, we compared the characteristics of women consenting to participate in the study to all women diagnosed with epithelial ovarian cancer using New Jersey State Cancer Registry data in the same counties during a similar time period [32]. Race and ethnic distribution was similar, while the cases consenting tended to be younger, with a median age of 56 years at diagnosis, compared to a median age of 61 years at diagnosis for the total population of cases. The distribution by histology, stage, and grade was generally similar. For controls, like most epidemiologic studies, we do not have information on those who could not be reached or did not participate. However, the distribution of the main risk factors for ovarian cancer in cases and controls is similar to that reported in other studies. Furthermore, non-response bias would only affect the study validity if willingness to participate is related to the factors under evaluation [31,33]. The possible role of dietary factors in the etiology of ovarian cancer is not well known, and even less so that of phytoestrogens and soy foods. This reduces the possibility of response bias. Also, the fact that our results are in agreement with the current literature in this area lessens even further the concern over possible non-response bias.

Our sample size was relatively small and we may have lacked power to detect a significant association. Nevertheless, our results were in general agreement with other studies. A major strength of our study is that it was specifically designed to evaluate the association between phytoestrogen intake from foods and supplements and ovarian cancer risk. For example, we expanded the questionnaire to include important sources of these compounds. We also computed all the major phytoestrogens for a detailed assessment.

Future studies should conduct a comprehensive assessment by including all major sources of phytoestrogens (including a detailed assessment of phytoestrogens in supplements) and should have sufficient power to evaluate the association, preferably using a prospective design, which avoids the major biases inherent to the case-control approach (such as recall and selection bias).

## Conclusions

Although comparison of results across studies is challenging due to differences in methods and study populations, our findings, together with the few studies published in this area, suggest that soy and phytoestrogen may decrease ovarian cancer risk.

## Competing interests

The authors declare that they have no competing interests.

## Authors' contributions

EVB wrote the first draft of the manuscript. EVB and SHO conceptualized the study design and provided overall supervision for conducting the study. EVB, MK, and UC conducted all the data analyses. LEP implemented case ascertainment. LRR provided clinical expertise. All authors revised the article critically for important intellectual content and approved the final version of the manuscript.

## Appendix 1 - Additional food items, not included in the Block Food Frequency Questionnaire (version 98.2)

Soy yogurt

Frozen soy yogurt

Soy ice cream

Soy cheese

Soy hot dogs/cold cuts

Other soy meat substitutes

Cooked soybeans or edamame

Roasted soy nuts

Tempeh

Miso soup

Alfalfa sprouts

Soybean sprouts

Protein powders made from soy protein isolate

Soy milk *(included in the Block FFQ but it was included again in the supplemental foods page)*

Green tea

Garbanzo beans, chick peas, ceci beans or hummus

Dried fruit (e.g., apricots, raisins, prunes)

Seaweed

Sunflower seeds

Black licorice

## Pre-publication history

The pre-publication history for this paper can be accessed here:

http://www.biomedcentral.com/1472-6874/11/40/prepub
